# Bonding to silicate ceramics: Conventional technique compared with a simplified technique

**DOI:** 10.4317/jced.53570

**Published:** 2017-03-01

**Authors:** Juan-Luis Román-Rodríguez, Jorge-Alonso Perez-Barquero, Eva Gonzalez-Angulo, Antonio Fons-Font, Jose-Luis Bustos-Salvador

**Affiliations:** 1 Associate Lecturer, Department of Dental Medicine, Prosthodontic and Occlusion Teaching Unit, University of Valencia, University of Valencia General Studies (UVGS), Spain; 2 Assistant Lecturer, Department of Dental medicine, Prosthodontic and Occlusion Teaching Unit, UVGS, Spain

## Abstract

**Background:**

Silicate ceramic bonding is carried out by acid-etching with hydrofluoric acid (HF) followed by an application of silane. By replacing HF with ammonium polyfluoride, contained in the same flask as the silane, the number of steps in this clinical procedure, can be reduced, while maintaining bond strength values, and reducing toxicity. A shear bond test was performed to compare the conventional and the simplified surface treatment techniques.

**Material and Methods:**

Twenty ceramic samples were fabricated from IPS emax CAD® ceramic (Ivoclar Vivadent) and divided into two groups (G1 and G2) (n=10). The conventional technique was applied to G1 samples, and the simplified technique to G2 samples. A resin cement cylinder was bonded to each sample. Afterwards, samples underwent shear bond strength testing in a universal test machine.

**Results:**

G1 obtained 26.53±6.33 MPa and G2 23.52±8.41 MPa, without statistically significant differences between the two groups.

**Conclusions:**

Monobond Etch&Prime appears to obtain equivalent results in terms of bond strength while simplifying the technique. Further investigation is required to corroborate these preliminary findings.

** Key words:**Shear bond strength, surface treatment, bonding to ceramic, hydrofluoric acid, ammonium polyfluoride.

## Introduction

Silicate ceramic-based esthetic restorations are bonded following a procedure that requires both mechanical (hydrofluoric acid - HF) and chemical (silane) surface treatment (1,2). But recently a new product has been introduced that replaces HF with ammonium polyfluoride and also contains silane, so that it combines the two surface treatments and simplifies the bonding procedure (Monobond Etch & Prime®; Ivoclar Vivadent, Schaan, Liechtenstein). The conventional bonding technique for silicate ceramics needs more steps and a longer time than this new simplified technique ( Table 1).

**Table 1:**
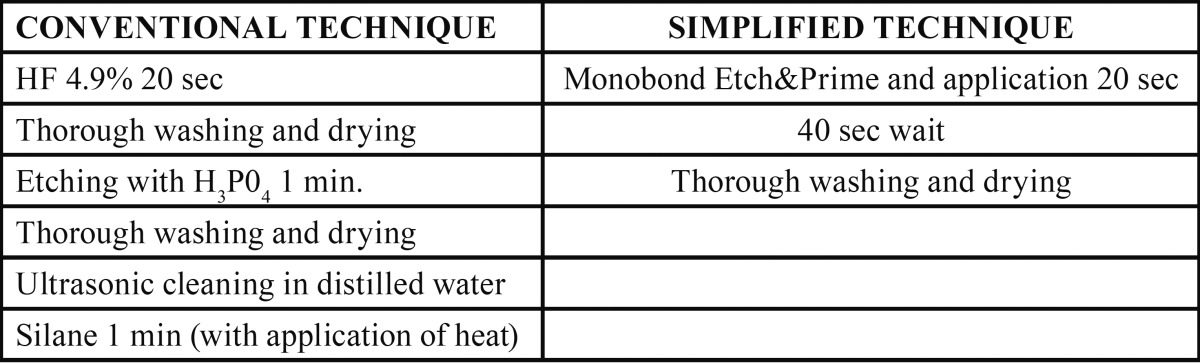
Detailed description of all steps in surface treatment techniques of silicate ceramics. H3P04= orthophosphoric acid.

Furthermore, it has been shown that ammonium polyfluoride is more biocompatible and safer than HF.

The working hypothesis of this study was that the simplified technique (Monobond Etch & Prime) would produce equivalent shear bond strength values to the conventional technique. To test this hypothesis, the shear bond strengths produced by the two techniques were compared.

## Material and Methods

Twenty ceramic samples were fabricated from IPS emax CAD® ceramic (Ivoclar Vivadent) and divided into two groups (G1 and G2) (n=10). The conventional technique was applied to G1 samples, and the simplified technique to G2 samples. A resin cement cylinder was bonded to each sample using ExcITE® adhesive (Ivoclar Vivadent) and Variolink II® luting agent (base and catalyzer) (Ivoclar Vivadent) ( Table 2). Afterwards, the samples were stored for 24 hours at 37º in a humid atmosphere. They then underwent shear bond strength testing in a Shimadzu® AGX 100 KN universal test machine. Statistical analysis of the data applied the Mann-Whitney non-parametric test (*p* <0.05).

**Table 2:**
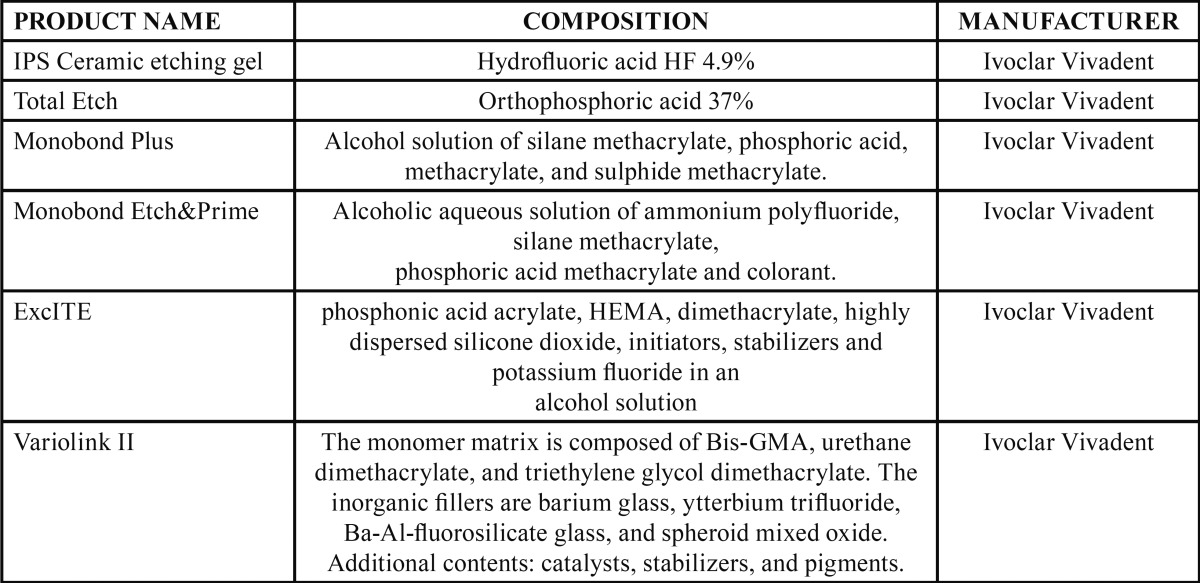
Product compositions.

## Results

**Figure 1:**
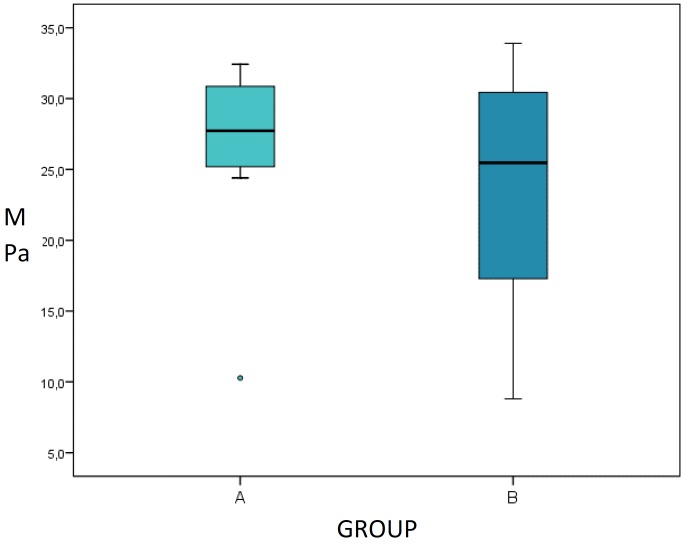
Box Plot showing similar median ranges, but with greater dispersion of G2 data.

In the shear bond strength test, G1 obtained 26.53±6.33 MPa and G2 23.52±8.41 MPa, without statistically significant differences between the two groups ( Table 3, Fig. 1). In both groups, 80% of failures were mixed (adhesive and cohesive), situated in the resin cylinder, and the remaining 20% adhesive failures; no purely cohesive failures were observed.

**Table 3:**
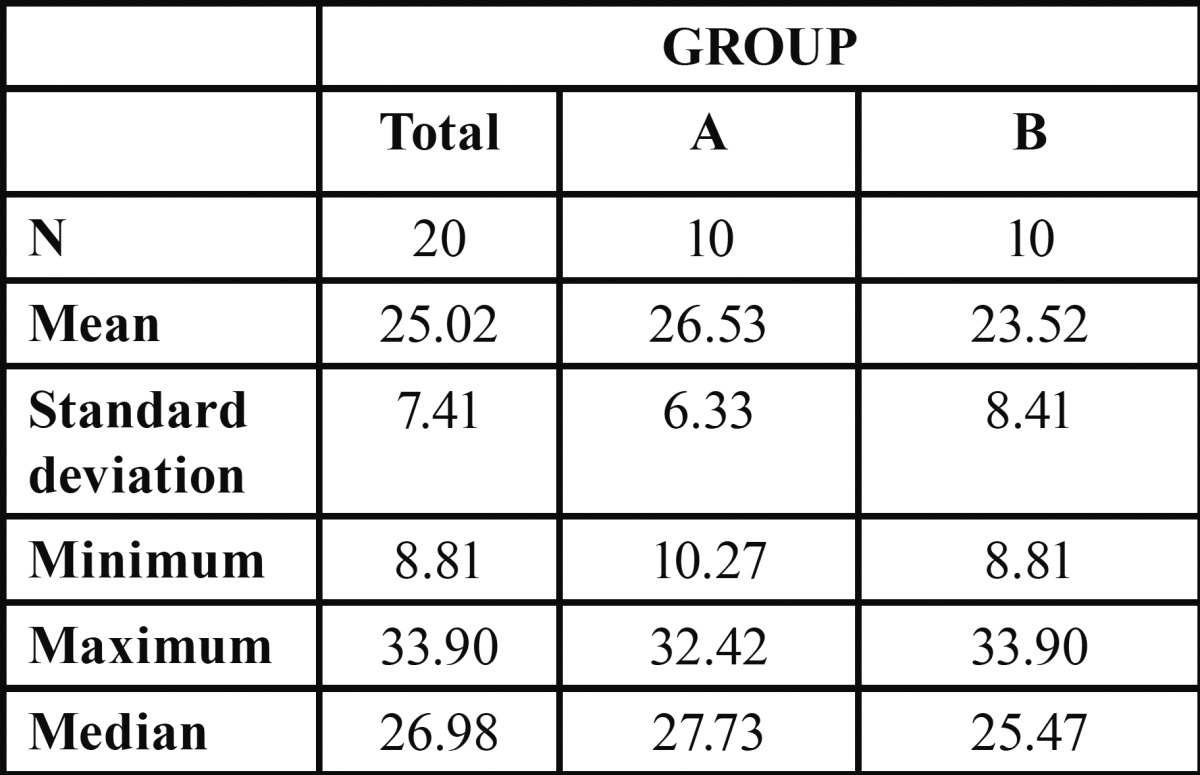
Shear bond strength test: descriptive statistics.

## Box Plot showing similar median ranges, but with greater dispersion of G2 data. 

When a ceramic restoration debonds, the resin cement usually remains on the restoration (3), which demonstrates that the ceramic-to-silicate cement bond is more consistent than the bond between cement and tooth. When a new product appears on the market that simplifies the bond procedure to ceramic, and shows predictable results, it must be fully investigated to ensure that the technique does not involve a loss of bond strength.

In dental ceramic bonding procedures, different surface treatments are used, both mechanical and chemical, which aim to maximize adhesion. These include sandblasting, silica coating, the application of different acids, different types of monomers or silane, but none of them have proved as effective as HF followed by silanization (4,5).

It has been observed that the use of H3PO4 eliminates the microprecipitates that appear after HF etching, while ultrasonic cleaning eliminates the macroprecipitates. When treating feldspathic ceramic surfaces, it is necessary to apply both processes, although in the case of HF-etched silicates with a lower presence of precipitates, the use of H3PO4 and ultrasonic cleaning is optional, and so the number of steps in the conventional procedure can be reduced (6). But it is still necessary to apply silane after HF. But when Monobond Etch&Prime is used, these two steps are reduced to a single application, which appears to obtain equivalent results in terms of bond strength while simplifying the technique.

The present results showed greater data homogeneity among G1 samples than G2, although G2 did obtain adequate bond strength values. Nevertheless, it is necessary to complement these findings with microtension testing and sample thermocycling.

Within the limitations of this *in vitro* study, it was found that the study’s working hypothesis was confirmed: the simplified technique with Monobond Etch&Prime may be used for bonding ceramic restorations with composite resins without compromising bond strength.

-Clinical relevance 

Silicate ceramic bonding technique by acid-etching with hydrofluoric acid (HF) followed by an application of silane can be simplified by replacing HF with ammonium polyfluoride, contained in the same flask as the silane. This simplified technique with Monobond Etch&Prime can be introduced clinically reducing the number of steps in the procedure, while maintaining bond strength values, and reducing toxicity.
